# Recent Advances in CRISPR/Cas9-Mediated Genome Editing in *Dictyostelium*

**DOI:** 10.3390/cells8010046

**Published:** 2019-01-12

**Authors:** Tetsuya Muramoto, Hoshie Iriki, Jun Watanabe, Takefumi Kawata

**Affiliations:** Department of Biology, Faculty of Science, Toho University, 2-2-1 Miyama, Funabashi, Chiba 274-8510, Japan; 5215015i@nc.toho-u.ac.jp (H.I.); 6217020w@st.toho-u.ac.jp (J.W.); tkawata@bio.sci.toho-u.ac.jp (T.K.)

**Keywords:** CRISPR/Cas9, CRISPR vector, *Dictyostelium*, genome editing, tRNA-based expression, Amoebozoa

## Abstract

In the last 30 years, knockout of target genes via homologous recombination has been widely performed to clarify the physiological functions of proteins in *Dictyostelium*. As of late, CRISPR/Cas9-mediated genome editing has become a versatile tool in various organisms, including *Dictyostelium*, enabling rapid high-fidelity modification of endogenous genes. Here we reviewed recent progress in genome editing in *Dictyostelium* and summarised useful CRISPR vectors that express sgRNA and Cas9, including several microorganisms. Using these vectors, precise genome modifications can be achieved within 2–3 weeks, beginning with the design of the target sequence. Finally, we discussed future perspectives on the use of CRISPR/Cas9-mediated genome editing in *Dictyostelium*.

## 1. Molecular Genetics Approaches in *Dictyostelium*

The social amoeba *Dictyostelium* is a microbial model organism widely used to understand cellular and developmental biology. Nutrient depletion drives cells to aggregate and then form multicellular fruiting bodies. Their growth and development occur at room temperature under atmospheric CO_2_ levels; therefore, no special incubator is required. Although it lacks the complexity of metazoan model organisms, such as *Caenorhabditis elegans* and *Drosophila melanogaster*, *Dictyostelium* shares simple developmental processes with these organisms, including cell differentiation processes, pattern formation and morphogenetic cell movements. *Dictyostelium* cells are also used to understand fundamental cellular functions that are affected in human diseases, including cell motility, phagocytosis, macropinocytosis and chemotaxis.

The 34-Mb genome of *Dictyostelium discoideum* encodes approximately 12,500 predicted proteins [1]. The genomic sequence data suggest that *Dictyostelium* is a member of the Amoebozoa group diverted from the animal lineage before fungi, whereas it has many proteins that were previously considered metazoan-specific. For example, *Dictyostelium* possesses a phosphotyrosine-SH2 signalling pathway that is not present in yeast [2,3]. Additionally, 24 kinase subfamilies present in both *Dictyostelium* and Metazoa were apparently lost in the lineage of fungi [4].

To analyse the functions of these homologous proteins, powerful molecular genetics approaches are available for *Dictyostelium*, including the expression of GFP- or epitope-tagged proteins, gene knockout via homologous recombination, insertional mutagenesis and gene silencing. Because *Dictyostelium* is a haploid organism, knockout mutants via homologous recombination can be generated in a short period of time. Genes encoding myosin heavy chain and alpha-actinin were successfully disrupted in 1987, providing insights into the physiological function of these proteins [5,6]. Subsequently, various efforts have been made to increase the efficiency of gene knockout [7,8,9,10,11]. Among these techniques, it should be noted that Paschke et al. developed faster methods to select transformants via growth on bacteria rather than axenic medium. Multiple knockout mutants were also generated using the Cre-loxP system, by which genes were sequentially knocked out by recycling the blasticidin cassette [12,13]. One remarkable example of multiple gene knockouts is the generation of a sextuple mutant lacking five PI3K genes and PTEN [14]. In addition to gene knockout, insertional mutagenesis using restriction enzyme-mediated integration (REMI) has been used to study many genes involved in cellular and developmental processes [15,16]. It is a relatively unbiased method to perform genome-wide forward genetic screening, and the resulting collections of mutants are used to analyse phenotypes via high-throughput screening [17]. Recently, by combining REMI and next-generation sequencing technology (REMI-seq), a large-scale gene knockout library was generated [18]. Another genetic approach for gene silencing involving the expression of antisense RNA or RNAi has been used for functional analysis, although its use is limited to specific experiments [19,20,21]. Transcription Activator-Like Effector Nuclease (TALEN) was reported as a simple genome editing technique in a number of model organisms [22,23,24], but there is no report that site-specific genome editing has been achieved in *Dictyostelium*. A major disadvantage is that the cloning of TALE repeats is technically challenging because of 15–20 repeat sequences. The generation of knockout construct involves simple cloning, and the efficiency of homologous recombination is high enough in *Dictyostelium*. In recent years, CRISPR/Cas9 technology has revolutionised biological research by permitting precise genome editing in many organisms, including *Dictyostelium* [25].

## 2. CRISPR/Cas9 System for *Dictyostelium*

**Figure 1:**
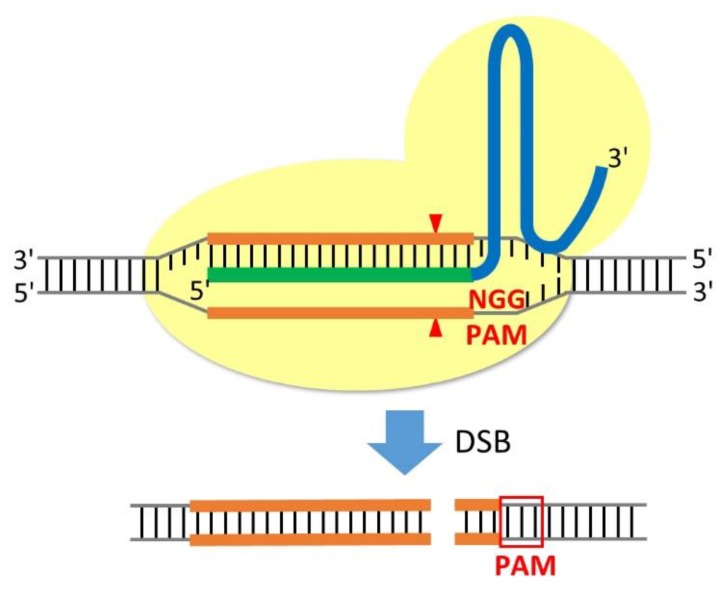
Schematic of RNA-guided Cas9 nuclease. Cas9 nuclease with a nuclear localisation signal (yellow) is recruited to genomic DNA by a chimeric single-guide RNA (sgRNA), containing a 20-nucleotide target sequence (green) and a universal tracrRNA (blue). The target sequence can be designed to target any genomic locus directly upstream of a short PAM sequence (red). Cas9 nuclease-mediated double-strand breaks (DSBs) occur approximately 3 bp upstream of the PAM sequence (red arrowheads).

CRISPR was developed from a prokaryotic adaptive immune system into a powerful genome editing technique. It requires two components: Cas9 nuclease with a nuclear localisation signal (NLS) and chimeric single-guide RNA (sgRNA) (Figure 1). In the widely used type-II CRISPR/Cas9 system, Cas9 nuclease, derived from *Streptococcus pyogenes* (SpCas9), recognises a 5′-NGG-3′ sequence in a specific protospacer-adjacent motif (PAM). The sgRNA is an artificial fusion RNA essential for CRISPR activity that contains a targeting sequence (crRNA) and a Cas9 nuclease-recruiting sequence (tracrRNA). By modifying a 20-nucleotide sequence at the 5′ end of sgRNA, Cas9 can be recruited to any desired gene of interest. After Cas9 induces site-specific double-strand breaks (DSBs), the cells repair the DNA damage via two mechanisms: non-homologous end-joining (NHEJ) and homology-directed repair (HDR). Repair via NHEJ may introduce short insertions and deletions (indels); therefore, frameshift mutations can arise and prevent functional protein expression. Frameshift mutations can also create premature stop codons within the middle of the targeted gene. Compared with NHEJ, the HDR pathway can insert tags or fluorescent proteins at genes of interest in a site-specific manner.

Designing guide RNA is an essential step in experiments because sgRNA is responsible for recruiting Cas9 nuclease to the specific gene region. There are two primary considerations in the selection of the target sequences, one of which is the presence of a PAM sequence in the locus. The target sequence must be adjacent to a PAM sequence. This limitation is not a severe issue for mammalian cells with GC-rich genomic sequences, in which the nucleotides appear on average every 8–12 bp [26]. However, NGG appears at a relatively lower frequency within the AT-rich *Dictyostelium* genome. This limitation is of particular concern when generating knock-in cells using HDR because the target site must be within the particular region to be edited. In addition to the PAM sequence requirement, the minimisation of off-target effects, which trigger unintended mutations within the genome, is the second consideration. To minimise off-target effects, the target sequence must be unique compared with the rest of the genome. Several web-based tools, including E-CRISP and Cas-Designer, exist for designing target sequences for *Dictyostelium*, with minimum off-target effects [27,28]. These tools require the selection of a user-defined PAM sequence, NGG in this case, the inputting of a gene of interest and the selection of a species, namely *Dictyostelium*. The programme gives a list of potential target sequences adjacent to PAM with several scores, which are calculated based on sequence homology and the number and positions of mismatches relative to the sgRNA sequence. Because the off-target sites have been experimentally validated [29], web-based tools, such as E-CRISP and Cas-OFFinder, computationally assess potential off-target sites for each intended target [28,30]. For effective targeting, we recommend designing at least two sgRNAs against the gene of interest and selecting highly specific sgRNAs, of which the 12 bp closest to the PAM sequence should match only one site within the whole *Dictyostelium* genome.

There are several more considerations in the selection of target sequences. Because the U6 RNA polymerase III promoter, which is used to express sgRNA in other systems, prefers guanine at the transcription start site to achieve effective expression, an extra guanine is recommended as the first base of the transcript [26,31]. However, the addition of the extra guanine is not suitable for our tRNA-based CRISPR/Cas9 expression system because it does not affect expression under the control of tRNA and sgRNA is cleaved at the tRNA-sgRNA junction during endogenous tRNA processing. Target sequences containing more than a four-thymidine repeat should be avoided because they are known as a termination signal for RNA polymerase III. For effective knockout, the target sequences are recommended to be selected within the first half of the gene because the targeting of 3′ exons may not result in the complete inhibition of gene function. The PAM sequence itself is absolutely required for cleavage, but it is not part of the sgRNA sequence; therefore, it should not be included in the sgRNA.

**Figure 2:**
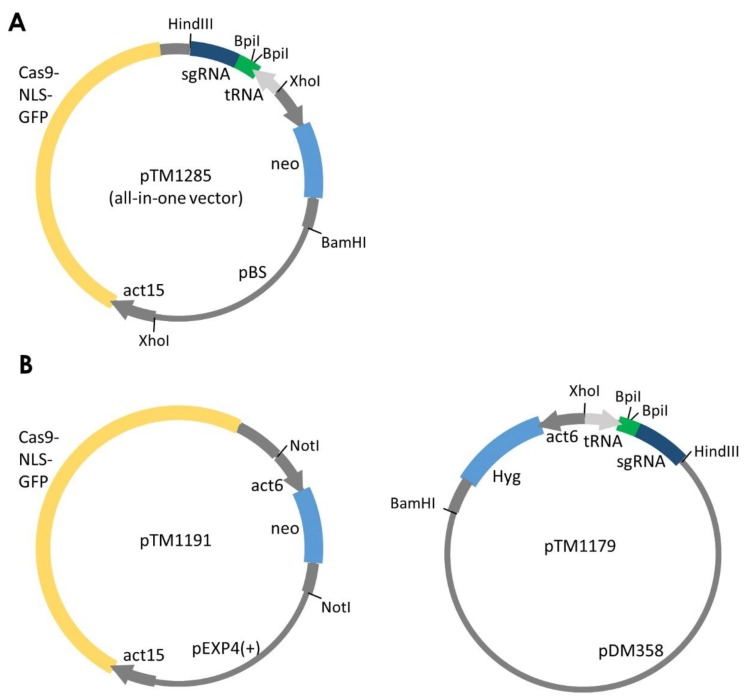
All-in-one and two-plasmid–based systems to express single-guide RNA (sgRNA) and Cas9. (**A**) Schematic of the all-in-one system expressing Cas9 nuclease and sgRNA. (**B**) Two-plasmid–based system for expressing Cas9 and sgRNA from two separate plasmids. The target sequence is cloned into these plasmids using BpiI sites. Both pTM1285 and pTM1179 can be used to express multiplex sgRNAs by inserting multiple tRNA-sgRNA modules. act15, act15 promoter; act6, act6 promoter; tRNA, isoleucine tRNA, neo, neomycin resistance gene; Hyg, hygromycin resistance gene.

We have developed two different CRISPR/Cas9-expressing systems: the all-in-one system and the two-plasmid–based system (Figure 2). These plasmids are available from NBRP Nenkin [32]. The all-in-one system was constructed by assembling tRNA-sgRNA, Cas9 nuclease and G418 resistance cassette into a pBluescript II vector. sgRNA expression is then induced by RNA polymerase III-dependent promoters of isoleucine tRNA. Because eukaryotic tRNA genes possess an internal promoter located within the transcribed region [33], the upstream region of tRNA is not contained in this vector. A six-thymidine transcription termination signal is inserted at the 3′ end of tracrRNA. Endogenous tRNA-processing machinery naturally cleaves sgRNA from the primary transcript, and the resulting sgRNA contains no extra nucleotides at either end. Transcription under the control of RNA polymerase II is unsuitable because an additional 5′ cap or 3′ poly-A tail could potentially inactivate the nuclease activity of the Cas9/sgRNA complex [34]. Furthermore, the mature mRNAs are transported from the nucleus to the cytoplasm; thus, Cas9/sgRNA complexes are isolated from the genomic DNA. The Cas9 nuclease contained in this vector was obtained from *S. pyogenes*, with optimisation of the initial 47-amino acid sequence for *Dictyostelium* codon usage [35]. Because the all-in-one vector contains no element for extra-chromosomal replication, transformed cells express the proteins transiently for a short period and then lose the vector during cell division. The transient expression successfully introduced indel mutations into the *Dictyostelium* genome, in which >70% efficiency for gene targeting was exhibited. The all-in-one system was also used to create multiple gene knockouts. Multiple tRNA-sgRNA modules subcloned into the final destination vector, pTM1290 [25], can be ligated to pTM1285 via digestion with XhoI and HindIII to express multiple sgRNAs. Plasmids expressing five PI3K sgRNAs were generated to simultaneously target genes with a high efficiency of 73–100%. One of the advantages of using transient expression vectors is that the drug resistance marker is not integrated into the genome; therefore, subsequent experiments using a drug marker can be performed.

In the two-plasmid–based system, Cas9 nuclease and sgRNA were separated into two different plasmids, which were finally combined inside the cells to form Cas9/sgRNA complexes. The first plasmid was used to express Cas9 nuclease with a G418 resistance cassette, whereas the second plasmid contained tRNA-sgRNA with a hygromycin resistance cassette. Therefore, transformed cells are resistant to both G418 and hygromycin, permitting the use of only blasticidin as a drug resistance marker for further genetic manipulation. Upon the co-expression of the two plasmids, the gene encoding tdTomato was targeted (99.4%, n = 1618). Thus, the two-plasmid–based system exhibited extremely high efficiency for genome editing in *Dictyostelium*. However, the stable expression of the plasmids under the control of a drug resistance cassette is believed to increase the risk of off-target effects. This expression system may not be suitable for inducing indel mutations because of the off-target effects, but it should be useful for other applications, such as transcriptional activation/repression, epigenetic modification and genomic imaging based on dCas9, which lacks nuclease activity [36,37]. 

## 3. Comparison of CRISPR/Cas9-Mediated Genome Editing in Microorganisms

A number of studies have provided evidence for successful CRISPR-mediated genome editing in microorganisms, such as *Trypanosoma cruzi*, *Plasmodium falciparum*, *Phaeodactylum tricornutum*, *Phytophthora sojae*, *Chlamydomonas reinhardtii*, *D. discoideum*, and *Saccharomyces cerevisiae* (Table 1) [25,38,39,40,41,42,43]. A popular way to prepare Cas9 and sgRNA in vivo is direct expression from all-in-one vectors. Since these microorganisms are phylogenetically separated from animals, codon-optimised SpCas9 to each species rather than adapting to human codon usage was often used. The most commonly used sgRNA expression system is RNA polymerase III-dependent U6 promoter because expression of short hairpin RNAs (shRNAs) driven by U6 promoter is well optimised for gene silencing in mammalian cells [44]. In the system, efficient transcription is found through upstream sequences because U6 small nuclear RNA genes have promoters located entirely upstream of the gene. But in yeast, A box and B box that are required for interaction of transcription factor IIIC (TFIIIC) are located within and downstream of the gene, respectively [45]; resulting efficient transcription is not observed via upstream sequence alone. Thus, snoRNA SNR52 promoter was used to express sgRNA in yeast [38]. Likewise, U6 promoter was attempted to be used in *D. discoideum* and *P. sojae*, but it was found that sufficient expression could not be obtained [25,39]. In these organisms, endogenous tRNA-processing machinery for expression sgRNA or ribozyme-based method to release mature sgRNA were used instead of the commonly used U6 promoter. Since tRNA and its processing system are well conserved in all organisms, the sgRNA expression system is expected to be used in a wide range of species where CRISPR-mediated genome editing has not been demonstrated. The social amoebae *Dictyostelium* is a member of the Amoebozoa, but to date, there has been no report on the success of CRISPR-mediated genome editing in the pathogenic amoeba *Acanthamoeba* and *Entamoeba*. Because a few expression vectors have been developed to express foreign and endogenous genes in both *Acanthamoeba* and *Entamoeba* [46,47,48], our system for generating gene knockout can be applied to these species. tRNA-based expression system also has the advantage of being able to disrupt multiple genes simultaneously via expression of multiplex sgRNA from a single expression vector [25,49,50,51]. The ribozyme-based method can also produce multiplex sgRNA [52]. One of the advantages of this technique is that the concentration of each sgRNA becomes equal due to expression from the same promoter. Although drawback of RNA polymerase III-dependent promoters lacks the ability to express in a tissue-specific manner to achieve tissue-specific genome editing, the ribozyme-based sgRNA production can be used as tissue-specific promoters or promoters regulated by environmental signals because the primary transcripts are automatically processed to release sgRNAs.

## 4. Construction of sgRNA-Expressing Plasmids and Isolation of CRISPR/Cas9-Mediated *Dictyostelium* Cell Lines

**Figure 3:**
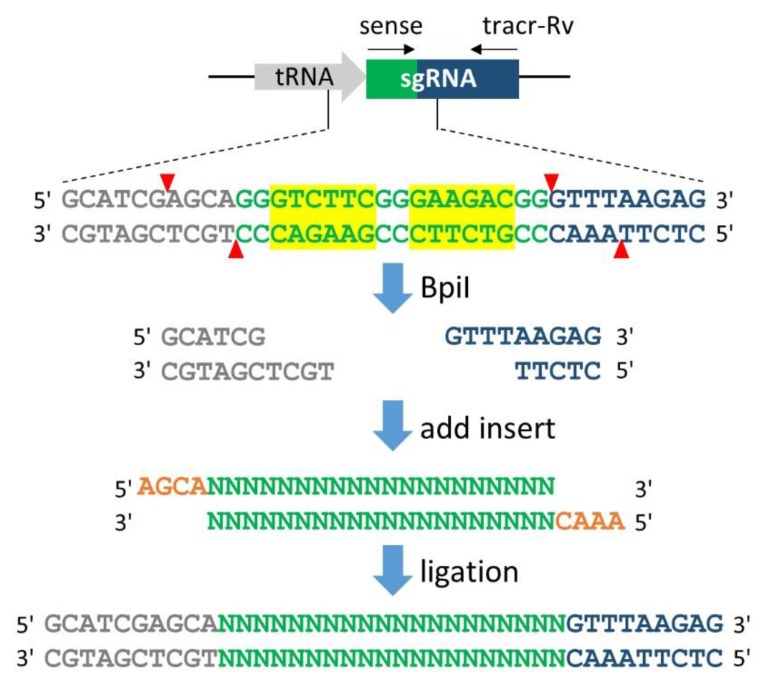
Generation of the single-guide RNA (sgRNA) expression vector. A pair of annealed oligonucleotides is easily cloned into the tRNA-sgRNA junction via BpiI restriction sites. The overhang sequences 5′-AGCA-3′ (orange) for the sense oligonucleotide and 5′-AAAC-3′ (orange) for the antisense oligonucleotide are compatible with the ends of BpiI-digested overhangs. The sense oligonucleotide is the 20-nucleotides target sequence preceding 5′-NGG-3′ (PAM sequence) in genomic DNA; thus, the PAM sequence should not be included in the oligonucleotide. After mixing the two oligonucleotides in equal molar amounts, the mixture is heated at 95 °C for 5 min and slowly cooled to 25 °C (1 °C/min) using a thermal cycler. The annealed oligonucleotide pairs are then ligated into the BpiI-digested vector via the Golden Gate digestion/ligation reaction performed in a thermal cycler using five cycles of 37 °C for 5 min and 16 °C for 15 min. After the cycle is finished, an additional BpiI digestion is performed at 37 °C for 60 min to prevent the contamination of the non-integrated vector before bacterial transformation. Black arrows indicate primers used for colony PCR, sense; target sense oligonucleotide, tracr-Rv; 5′-AAGCTTAAAAAAAGCACCGACTCGGTGCC-3′. Yellow boxes indicate BpiI recognition sites and red arrowheads denote their cleavage sites. Grey, green and blue denote tRNA, the target sequence and tracrRNA, respectively.

The all-in-one CRISPR/Cas9 vector and the two-plasmid–based system contain two BpiI sites after the tRNA gene. Because BpiI is a type IIS restriction enzyme that recognises asymmetric nucleotide sequences (i.e., GTCTTC or GAAGAC) and cleaves outside the recognition site, the truncated ends can be ligated to the target sequence without the addition of extra nucleotide. To clone the 20-nucleotide target sequence into the BpiI-digested expression vectors, two oligonucleotides with 4-nucleotide overhangs that are compatible with the ends of BpiI-digested vectors are synthesised (Figure 3). Because the correctly inserted clones lose the BpiI sites, the resulting vector is no longer digested by BpiI. The correct integration of the target sequence into the vector is assessed by colony PCR using the following primers: the sense oligonucleotide and tracr-Rv. For further validation, it is recommended to digest the vectors using BpiI because correct integration removes the BpiI site, followed by sequence analysis via conventional Sanger sequencing using the tracr-Rv primer.

The manipulation of multiple genes by CRISPR/Cas9 is rapid and efficient compared with homologous recombination using the Cre-loxP system. We generated a multiplex sgRNA expression system to express tandem repeats of tRNA-sgRNA from one vector, resulting in the simultaneous expression of 2–20 sgRNAs [25]. To clone the multiple tRNA-sgRNA modules, we applied the Golden Gate method used in the Platinum Gate TALEN kit [53]. Although this cloning procedure is not as simple as one-step cloning for expression of single sgRNA in the all-in-one vector, simultaneous expression from the multiplex vector resulted in better genome editing efficiency than individual tRNA-sgRNA expressing vectors. When electroporation was performed using two independent sgRNA expression vectors and the same amount of DNA, the gene disruption efficiency was reduced to less than half of that when two sgRNAs were simultaneously expressed from one multiplex vector. Thus, it is valuable to employ multiplex sgRNA expression vectors to generate mutants containing three or more gene knockouts.

Efficient methods for the electroporation of the CRISPR/Cas9 vector and clonal isolation are indispensable for successful genome editing in *Dictyostelium*. The DNA used should be of high quality and should not exceed 10% of the total volume to achieve maximum results. Higher amounts of plasmid DNA (e.g., 10 µg) tend to produce more colonies than lower amounts (e.g., 1 µg), but the number of colonies obtained after electroporation is sufficient for further investigation in both cases. We use electroporation-based transient expression using H50 buffer [25]. Recently, the simple HEPES buffer H40 was reported to provide a similar efficiency as the complex H50 buffer [7]. We also confirmed its efficiency for the transient expression of CRISPR/Cas9 vectors. After 8–16 h of electroporation, the cells are maintained in HL5 medium containing 10 µg/mL G418. The cells are collected within 1–3 days before becoming round in shape and then plated on SM agar plates with *K. planticola*. Approximately 4 days later, individual plaques on the plates are used for further PCR analysis. If the CRISPR vector remains in the cells, genome editing could continuously occur and off-target effects are enhanced; thus, it is recommended to confirm that the cells are sensitive to G418.

The most common method for analysing genome modification is PCR amplification of the predetermined target region. In this case, a well-designed and optimised PCR condition is essential for precise results. For successful PCR, we recommend the use of an appropriate annealing temperature and appropriate primer concentrations using the wild-type genome before mutated clones are grown. As one of the PCR primers is designed to span the Cas9 cleavage site, PCR does not amplify most of the indel mutants. However, even if there is a deletion or insertion in the target site, the possibility that inefficient PCR amplification may be observed cannot be excluded. To minimise false-positive amplification, it is essential to use a high-fidelity DNA polymerase, such as KOD plus (TOYOBO) or PfuUltra (Agilent). Indel mutations can also be detected through Sanger sequencing, by which purified PCR products including the target region are cloned into TA cloning vectors.

To indel mutants from SM agar plates, we usually collect approximately 20 clones as well as one control clone that is not targeted in the gene and extract genomic DNA using lysis buffer containing proteinase K. It is crucial to obtain high-quality DNA for better PCR results. The incorporation of an excess amount of bacteria from SM agar plates into the lysis buffer sometimes interferes with the accuracy of PCR. In the mutation detective PCR, the amplification efficiency at the Cas9 cleavage region is compared between CRISPR-mediated mutants and the control clone that is not targeted in the gene. Although we found that more than half of the transformants contained indel mutations [25], this ratio can differ depending on the gene of interest. Even if the gene is essential for proliferation, it is highly possible to obtain mutated cells in which three or six nucleotides were inserted or deleted in the target region, resulting in the insertion or deletion of one or two amino acids in the protein. Indeed, mutations featuring the insertion of three or six nucleotides were obtained in five PI3K mutants through selection in axenic medium [25]. In addition, a six-nucleotide insertion was detected in a gene knockout involved in metabolism. The maintenance and selection of cells with bacteria [7] rather than axenic medium could be one solution to obtain macropinocytosis-defective mutants.

It is recommended to compare and analyse several CRISPR-mediated clones for functional analysis to minimise the risk of off-target effects. In fact, however, off-target effects were not more frequent than expected in vivo. Recognition of the appropriate target site by sgRNA in the CRISPR/Cas9 system is sufficiently specific, as it has been reported that cleavage activity is decreased when a single nucleotide mismatch is present within the sgRNA sequence in mammalian cells [54]. Indeed, there was no mutation at potential off-target sites in CRISPR-mediated mutant mice according to the rule [55]. Furthermore, because we use a transient expression system, genome editing does not continuously occur. However, it cannot be said that there are no off-target effects in *Dictyostelium*, and the analysis of multiple mutants is recommended. 

## 5. Prospects

Since developments in the field of gene mutagenesis in the 1980s, specifically in *Dictyostelium*, several methods have been introduced such as gene overexpression, homologous recombination-mediated gene knockout and RNAi. Recently, the development of CRISPR/Cas9-mediated gene mutagenesis has revolutionised the field of molecular biology. Genome editing in *Dictyostelium* remains in the developmental stage, but it is certainly one valid option for genetic analysis. Additional work is needed to improve the genome editing protocols for gene manipulation.

**Figure 4:**
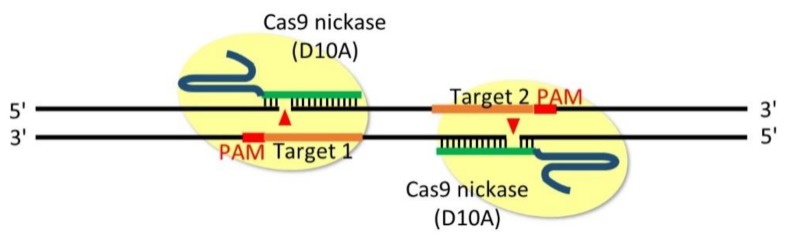
Double-nicking method that can introduce insertion/deletion (indel) mutations between a pair of single-guide RNAs (sgRNAs). The Cas9 D10A nickase generates a nick (arrowheads) in the strand complementary to the sgRNAs. The orange line denotes the pair of sgRNAs targeting opposite strands of the genome, and red denotes the PAM sequences. PAM sequences with an outward orientation generate 5′ overhangs, which are necessary for the robust induction of indel mutations.

Most importantly, off-target effects represent the main limitation. Recently, a double-nicking method based on the Cas9 D10A nickase mutant, which can reduce off-target effects 50–1500-fold, has been developed [56,57]. Cas9 nickase is capable of creating single-strand breaks (nick) instead of DSBs as caused by Cas9 nuclease (Figure 4). Because individual nicks in the genome are precisely repaired by base excision repair, DSBs only occur when simultaneous nicking is generated via recruiting a pair of offset sgRNAs targeting opposite strands of genomic DNA. Thus, it is possible to generate DSBs by nicking two proximal sites (generally 0–20 bp apart) with PAM sequences facing outward to leave 5′ overhangs. However, the design of two sgRNAs targeting sites separated by 20 bp is generally difficult because the GC-rich NGG PAM sequence does not frequently appear in the *Dictyostelium* genome. It is relatively easy to design two sgRNAs targeting sites separated by approximately 100–200 bp even in the AT-rich *Dictyostelium* genome, whereas a previous report suggested that the frequency of indel mutations is decreased when the offset distance is approximately 100 bp [57]. It should be noted that the generation of 3′ overhangs instead of 5′ overhangs did not lead to a robust NHEJ-mediated indel mutation as previously reported [56]. The 5′ overhangs also represent a limitation in designing two sgRNAs with a short offset distance in *Dictyostelium*.

One potential solution to increase targetable loci is a Cas9 variant with altered PAM sequences. Cas9 from *S. pyogenes* (SpCas9) is the most common choice for genome editing, but the use of a short NGG as the PAM sequence is restricted in AT-rich genomes. Recently, several Cas9 variants with altered PAM sequences were reported, such as NGA (SpCas9 VQR), NGAG (SpCas9 EQR), NGCG (SpCas9), NG, GAA, GAT (xCas9) and NG (SpCas9-NG) [58,59,60]. In addition, several Cas9 homologues derived from other species have been demonstrated to exhibit distinct PAM specificities, such as *Staphylococcus aureus* Cas9 (SaCas9), KKH SaCas9, *Lachnospiraceae bacterium* Cas12a (LbCas12a), *Acidaminococcus sp*. (AsCas12a) and *Streptococcus canis* (ScCas9), which recognise NNGRR, NNNRRT, TTTN, TTTN and NNG, respectively [59,61,62,63,64]. Replacing SpCas9 in our CRISPR vector with these various Cas9 homologues has a potential to mediate site-specific targeting that is not possible using conventional SpCas9. It is, therefore, possible to take advantage of the site specificity to perform Cas9 nickase-mediated site-specific gene knock-in using HDR and base editing. Base editing is known as a precise and straightforward genome editing technology that replaces targeted nucleotides without generating DSBs and homologous sequences [65]. The base editors reported previously only allowed the conversion of cytosine to thymine [66,67], but a new base editor, the adenosine base editor (ABE), can also convert adenine to guanine [68]. By applying these techniques, there is a possibility to generate site-specific modifications to a gene rather than inducing indel mutations in *Dictyostelium*, and they can be used for analysing essential genes by introducing point mutations in the functional domain.

Although CRISPR/Cas9-mediated genome editing is promising in *Dictyostelium*, there is room for improvement of the already efficient protocol. The current all-in-one vector has two BpiI sites, which are used to insert the target sequence between tRNA and sgRNA. If a BpiI site is present in the designed target sequence, this vector cannot insert the sequence via the Golden Gate digestion/ligation reaction. As we have constructed an all-in-one vector that possesses two sites for Esp3I (BsmBI), another type IIS restriction enzyme, a target sequence containing a BpiI site can be cloned into the tRNA-sgRNA junction via Golden Gate cloning. In addition, to obtain mutants that are deficient in axenic proliferation, such as those with macropinocytosis defects, it is necessary to perform selection using bacteria rather than axenic medium; therefore, we have generated an all-in-one vector in which the drug resistance marker is driven by the *coaA* promoter and terminated using the *mhcA* terminator. We confirmed that the new vector generated CRISPR-mediated indel mutants under bacterial selection. Several reports revealed that a genome-wide library of sgRNAs provides an opportunity to perform CRISPR-mediated forward genetic screening to identify genes involved in various functions, such as developmental processes and cellular functions [69,70,71,72]. Unlike the previously used forward genetic approaches, such as REMI mutagenesis or chemical mutagenesis, CRISPR-mediated forward genetic screening can be used to create mutant libraries of genome-wide or subpooled (e.g., kinase or nuclear proteins) genes.

## Figures and Tables

**Table 1:** Delivery system for CRISPR/Cas9-mediated genome editing in microorganisms.

Organism Name	Target Genes	Cas9 Protein	sgRNA Expression	Reference
*Trypanosoma cruzi*	*TcPFR1*, *TcPFR2*, *TcGP72*	human codon-optimised SpCas9 driven by ribosomal promoter	*T. cruzi* ribosomal promoter	[42]
*Plasmodium falciparum*	*gfp*, *kahrp*	human codon-optimised SpCas9 driven by *hsp* promoter	*P. falciparum* U6 promoter	[40]
*Phaeodactylum tricornutum*	*CpSRP54*	codon-optimised SpCas9 driven by *LHCF2* promoter	*P. tricornutum* U6 promoter	[43]
*Phytophthora sojae*	*Avr4/6*	human codon-optimised SpCas9 driven by Ham34 promoter	*P. sojae* Pol II *RPL41* promoter with ribozyme	[39]
*Chlamydomonas reinhardtii*	*hph*, *gfp*, *gluc*, *FKB12*	codon-optimised SpCas9 driven by CaMV 35S promoter	*Arabidopsis* U6 promoter	[41]
*Dictyostelium discoideum*	*acaA*, *pkaC*	partially codon-optimised SpCas9 driven by actin promoter	*D. discoideum* isoleucine tRNA	[25]
*Saccharomyces cerevisiae*	*CAN1*, *ADE2*, *LYP1*	human codon-optimised SpCas9 driven by Tef1 or Gal-L promoter	*S. cerevisiae* snoRNA SNR52 promoter	[38]
